# Supplementary figures and images for: NAD Supplement Alleviates Intestinal Barrier Injury Induced by Ethanol Via Protecting Epithelial Mitochondrial Function
Source: Nutrients. 2022 Dec 30;15(1):174. doi: 10.3390/nu15010174 (PMC9823589; doi:10.3390/nu15010174)

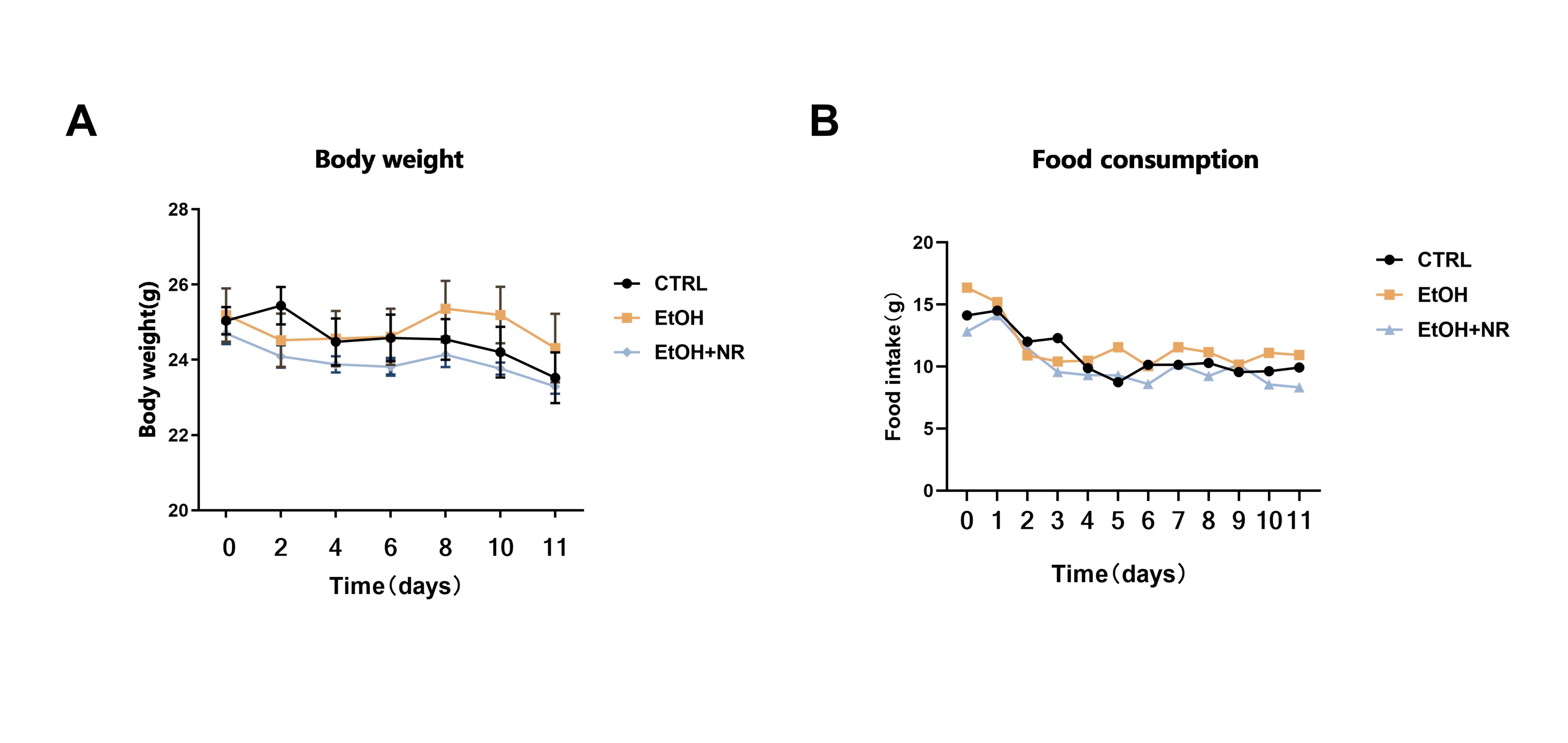

Supplement: Supplementary file 1 [file nutrients-15-00174-s001.zip › nutrients-1983700-supplementary.jpg]
